# Molecular investigations of *Mycobacterium tuberculosis* genotypes among baseline and follow-up strains circulating in four regions of Eswatini

**DOI:** 10.1186/s12879-023-08546-9

**Published:** 2023-08-29

**Authors:** Talent C. Dlamini, Brenda T. Mkhize, Clive Sydney, Nontuthuko E. Maningi, Lesibana A. Malinga

**Affiliations:** 1 Department of Medical Laboratory Sciences, Southern Africa Nazarene University, Manzini, Eswatini; 2 Biomedical and Clinical Technology, Department, Durban University of Technology, Durban, South Africa; 3 Microbiology Department, University of KwaZulu Natal, Durban, South Africa; 4 Department of Medical Microbiology, Faculty of Health Sciences, University of Pretoria, Pretoria, South Africa

**Keywords:** Tuberculosis, Multi-drug resistant tuberculosis, Drug sensitivity testing, Spoligotyping, Lineages, Genotypes

## Abstract

**Background:**

The tuberculosis (TB) epidemic remains a major global health problem and Eswatini is not excluded. Our study investigated the circulating genotypes in Eswatini and compared them at baseline (start of treatment) and follow-up during TB treatment.

**Methods:**

Three hundred and ninety (*n* = 390) participants were prospectively enrolled from referral clinics and patients who met the inclusion criteria, were included in the study. A total of 103 participants provided specimens at baseline and follow-up within six months. *Mycobacterium tuberculosis (M.tb)* strains were detected by GeneXpert® MTB/RIF assay (Cephied, USA) and Ziehl -Neelsen (ZN) microscopy respectively at baseline and follow-up time-points respectively. The 206 collected specimens were decontaminated and cultured on BACTEC™ MGIT™ 960 Mycobacteria Culture System (Becton Dickinson, USA). Drug sensitivity testing was performed at both baseline and follow-up time points. Spoligotyping was performed on both baseline and follow-up strains after DNA extraction.

**Results:**

Resistance to at least one first line drug was detected higher at baseline compared to follow-up specimens with most of them developing into multidrug-resistant (MDR)-TB. A total of four lineages and twenty genotypes were detected. The distribution of the lineages varied among the different regions in Eswatini. The Euro-American lineage was the most prevalent with 46.12% (95/206) followed by the East Asian with 24.27% (50/206); Indo-Oceanic at 9.71% (20/206) and Central Asian at 1.94% (4/206). Furthermore, a high proportion of the Beijing genotype at 24.27% (50/206) and S genotype at 16.50% (34/206) were detected. The Beijing genotype was predominant in follow-up specimens collected from the Manzini region with 48.9% (23/47) (*p* = 0.001). A significant proportion of follow-up specimens developed MDR-TB (*p* = 0.001) with Beijing being the major genotype in most follow-up specimens (*p* < 0.000).

**Conclusion:**

Eswatini has a high *M.tb* genotypic diversity. A significant proportion of the TB infected participants had the Beijing genotype associated with MDR-TB in follow-up specimens and thus indicate community wide transmission.

**Supplementary Information:**

The online version contains supplementary material available at 10.1186/s12879-023-08546-9.

## Introduction

Tuberculosis (TB) remains a major global health problem responsible for ill health among millions of people each year [1]. In the Kingdom of Eswatini, TB notification and incidence rates have been decreasing steadily in the past decade, from a peak incidence of 1,190 per 100,000 populations in 2009 to a low incidence rate of 329 per 100,000 populations in 2018 [2]. Notifications of drug resistant TB (DR-TB) mirrored those of drug-susceptible TB for the same period; however, DR-TB has not been adequately tracked by most TB programs in the Sub-Saharan Africa region, until recently [3]. The proportion of persons developing DR-TB while on treatment, including those with multidrug resistant TB (MDR-TB), increased over the years in Eswatini. It is important to establish, if resistance is due to high community transmission or arises *denovo* due to inadequate treatment or is due to both. MDR-TB, which is defined as resistance to at least isoniazid (INH) and rifampin (RIF) drugs used to treat TB diseases, is harder to diagnose, treat and is associated with unfavourable outcomes [4]. Moreover, DR-TB is not only indicative of failing TB patients and programs but is also more expensive for both [5]. Eswatini is committed to end TB and the then Prime Minister declared the disease a national emergency as far back as 2011 [6]. The study aims to characterize the genotypes of *Mycobacterium tuberculosis* (*M.tb)* genotypes collected from routine patients treated at four main regional centres in the Kingdom of Eswatini.

Eswatini clinical laboratories uses the sputum smear microscopy and GeneXpert® MTB/RIF assay (Cephied, USA) for the diagnoses of TB in presumptive patients. Furthermore, culture and drug sensitivity of the detected TB specimens is determined using the thin-layer agar, Löwenstein-Jensen and Bactec MGIT™ TB system methods. Of importance is that the detection of *M.tb* strain be identified for effective TB control strategies. Strain genotyping of circulating TB genotypes will help understand molecular epidemiology and transmission patterns. Advancements in molecular epidemiological techniques which have been reported to expand the ability to investigate and understand the TB epidemic are documented [7]. Spoligotyping remains the cost-effective method in resource limited regions with data shared in an international database. Notably, the genotyping molecular techniques are not conventionally used in resource-limited settings such as in Eswatini.

Eswatini is a small country that shares its borders with South Africa and Mozambique. There is a high burden of TB in Southern Africa region, especially in the three countries and information on genetic diversity of TB genotypes is important. Studies on circulating TB genotypes have been done in South Africa and Mozambique [8]. Currently there is lack information of circulating TB genotypes in Eswatini. Few studies have reported unique mutations and high rate of MDR-TB in Eswatini [9]. Both countries, South Africa and Mozambique have high TB genetic diversity and population in the region migrate due to increased economic activity between countries.

Hence, the current study investigated the circulating genotypes in Eswatini using the spoligotyping method and compared both baseline (start of treatment) and follow-up genotypic information with resistant data at six months or the last available positive specimen from patients on treatment.

## Materials and methods

**Fig. 1:**
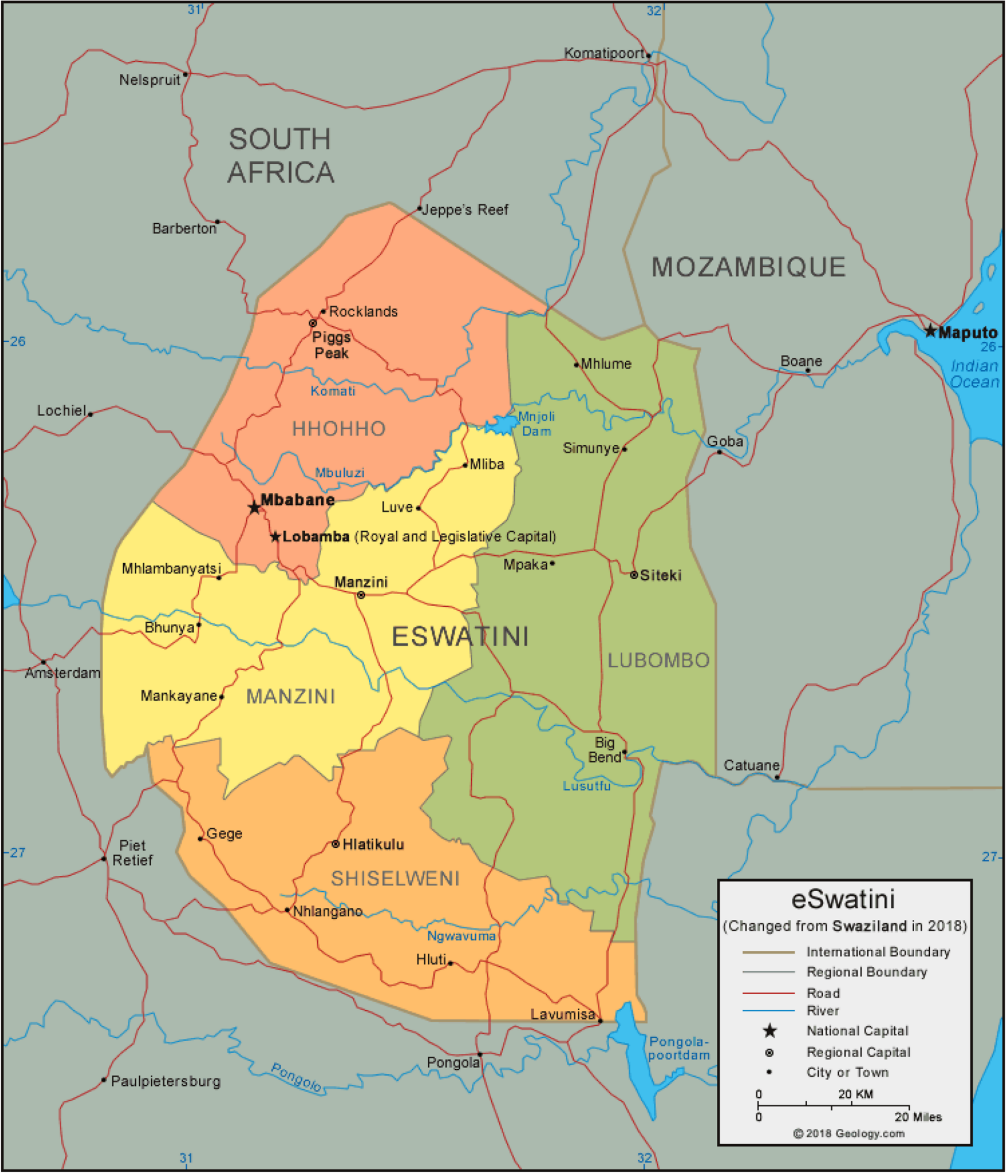
Eswatini map (Accessed on 2022 October 17, from: https://geology.com/world/swaziland-satellite-image.shtml)

The study prospectively recruited all presumptive TB patients from November 2017 to January 2019 seeking care at referral TB testing facilities from each of the four regions in Eswatini: Hhohho, Manzini, Lubombo and Shiselweni. These regions cover the entire Kingdom of Eswatini (Fig. 1).

### Ethical approval

Ethical clearance was obtained from Durban University of Technology Institutional Research Ethics Committee (IREC 111/17) and from Eswatini National Human and Health Research Review Board (MH/599C/ IRB 0009688/NHRRB012/16).

### Recruitment and enrolment

The study recruited presumptive TB patients between November 2017 and January 2019. Patients who presented with signs and symptoms suggestive of TB were enrolled into the study. Three hundred and ninety (*n* = 390) consenting adult participants, who were 18 years of age and above, as well being TB presumptive were approached for the study. A total of hundred and three participants (*n* = 103) that produced sputum for diagnosis were enrolled into the study and follow-up specimen collected for a minimum of two to six months. In addition, those who could not produce sputum on the day of recruitment even after the coughing officers’ intervention, as per the national guidelines, were provided with universal containers to take away for the collection of the sputum in the morning. The specimen was returned within three days after enrollment. Participants who did not meet the inclusion criteria were excluded from participating in the study.

### Baseline specimen processing

Baseline sputum specimens were processed for the detection of *M. tb* using the GeneXpert® MTB/RIF assay in a central laboratory in Mbabane according to manufacturer’s instruction (Cepheid). The specimen was decontaminated using *MycoPrep (*Becton Dickson*)*, and cultured on BACTEC Mycobacteria growth indicator tube (MGIT™) TB system (Becton Dickson, Sparks, Maryland). After a positive MGIT culture the Mycobacterium Protein 64 (MPT64) antigen assay was performed (SD Bioline), then phenotypic culture and drug sensitivity method were performed as previously published [10].

### Collection and processing of follow-up specimens

Follow-up specimens were collected between two and six months after processing of baseline specimens. Microscopy performed according to Zeihl-Neelsen method after decontamination. Both culture and drug susceptibility testing procedures were similar to baseline specimens.

### DNA extraction and spoligotyping

DNA was extracted using Genolyse (Hain Lifescience, Germany) from MGIT cultures and frozen at -20 °C at the University of Pretoria laboratory in South Africa. The spoligotyping was performed according to manufacturer’s instructions [11]. We used the SITVIT2 database (an updated version of SpolDB4) to assign TB genotypes to the major phylogenetic lineages and genotypes [10].

### Statistical methods

The genetic, genotype, and DST data sets were entered in a Microsoft Excel program. Then further analysed on Statistical Package for the Social Sciences (SSPS) version 25, Epi Info (version 3.5.1, 2008) and STATA 13.0 softwares. Chi square test were used to measure the level of association.

## Results

### Study population

**Fig. 2:**
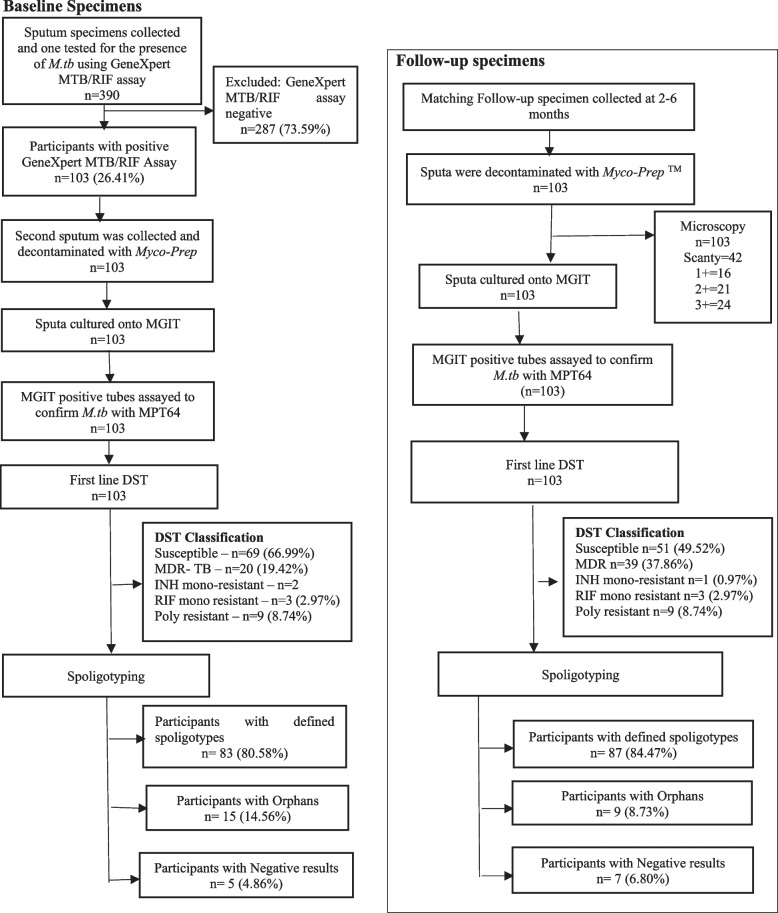
Participant flow diagram of baseline and follow-up specimens of patients collected at primary healthcare facilities in Eswatini. A total of *n* = 103 participants were enrolled into the study and followed through at 6th month

Of the 390 potential participants that were investigated between November 2017 and January 2019 for the presence of *M.tb*, 26.41% (103/390) had positive TB disease on at least one occasion (Fig. 2) (Table 1).

**Table 1:** Demographic data of four regions in Eswatini represented into the study

Demographic data	Characteristic	n (%)
Gender	Male	66 (64)
	Female	37 (36)
	TOTAL	103
Distribution per region	Manzini	56 (54.37)
	Hhohho	21 (20.39)
	Lubombo	13 (12.62)
	Shiselweni	13 (12.62)
	Total	103
Age distribution: Early adulthood	18—34	58 (56.31)
Early Middle adulthood	35—44	30 (29.13)
Late middle adulthood	45—64	14 (13.59)
Late adulthood	65 and above	1 (0.97)
	Total	103 (100)

### Drug susceptibility profile of the *M.tb* strains

The drug susceptibility testing (DST) results were generated at baseline (*n* = 103) and at follow-up, (*n* = 103). Although, most of the specimens at both baseline and follow-up (58.74%) were susceptible to all first-line anti-TB drugs, there was a significant proportion of specimens that were classified as RIF mono-resistant (28.16%). Furthermore, the poly-resistant specimens were 8.25% and those classified as INH mono-resistant were 4.85%. Notably, the region with a high proportion of MDR-TB specimens compared to the rest of the regions, was Lubombo, with 38.46%. Upon the analysis of the susceptibility patterns of the anti-TB drugs in all collected specimens (*n* = 206), it was revealed that most specimens had increased resistance to all the drugs at follow-up compared to baseline. Resistant specimens to RIF had significantly increased from 30.1% to 48.5% at follow-up (*p* = 0.005), with resistance to STR significantly increased from 27.2% to 39.8% (*p* = 0.038) and to EMB significantly increased from 21.4% to 36.9% (*p* = 0.011). Resistance to INH increased from 22.3% to 32.0%, however with no statistical significance (*p* = 0.079) (Table 2).

**Table 2:** The table shows DST pattern for all the first line drugs used in Eswatini per region

TB drugs	DST	Region					*p*-value	Baseline n (%)	Follow up n (%)	Total n (%)	*p*-value
		Hhohho n (%)	Manzini n (%)	Lubombo n (%)	Shiselweni n (%)	Total n (%)					
RIF	S	25 (59.5)	69 (61.6)	13 (50)	18 (69.2)	125 (60.7)	0.557	72 (69.9)	53 (51.5)	125 (60.7)	0.005*
	R	17 (40.5)	43 (38.4)	13 (50)	8 (30.8)	81 (39.3)		31 (30.1)	50 (48.5)	81 (39.3)	
	Total	42 (100)	112 (100)	26 (100)	26 (100)	206 (100)		103 (100)	103 (100)	206 (100)	
INH	S	31 (73.8)	82 (73.2)	15 (57.7)	22 (84.6)	150 (72.8)	0.197	80 (77.7)	70 (68.0)	150 (72.8)	0.079
	R	11 (26.2)	30 (26.8)	11 (42.3)	4 (15.4)	56 (27.2)		23 (22.3)	33 (32.0)	56 (27.2)	
	Total	42 (100)	112 (100)	26 (100)	26 (100)	206 (100)		103 (100)	103 (100)	206 (100)	
STR	S	28 (66.7)	74 (66.1)	16 (61.5)	19 (73.1)	137 (66.5)	0.853	75 (72.8)	62 (60.2)	137 (66.5)	0.038*
	R	14 (33.3)	38 (33.9)	10 (38.5)	7 (26.9)	69 (33.5)		28 (27.2)	41 (39.8)	69 (33.5)	
	Total	42 (100)	112 (100)	26 (100)	26 (100)	206 (100)		103 (100)	103 (100)	206 (100)	
EMB	S	29 (69)	79 (70.5)	17 (65.4)	21 (80.8)	146 (70.9)	0.639	81 (78.6)	65 (63.1)	146 (70.9)	0.011*
	R	13 (31)	33 (29.5)	9 (34.6)	5 (19.2)	60 (29.1)		22 (21.4)	38 (36.9)	60 (29.1)	
	Total	42 (100)	112 (100)	26 (100)	26 (100)	206 (100)		81 (78.6)	65 (63.1)	146 (70.9)	

*RIF* Rifampicin, *INH* Isoniazid, *STR* Streptomycin, *EMB* Ethambutol, *S* Susceptible, *R* Resistant

^*^p-value significant at *p* < 0.05

The drug susceptibility profiles were further analysed per region to determine the participant profiles among the different regions. Although most of the specimens in all the regions were susceptible to the four anti-TB drugs (Table 2), the Lubombo region had the highest drug resistance rates with all the anti-TB drugs tested. The resistance to RIF was 50% (*p* = 0.557), to INH was 42.3% (*p* = 0.197), to STR was 38.5% (*p* = 0.853) and to EMB was 34.6% (*p* = 0.639).

Notably, the analysed data suggests that a high proportion of participants infected with MDR-TB genotypes were from the Lubombo region. In addition, majority of these specimens in this region were resistant to all the anti-TB drugs.

### The distribution of the TB phylogenic lineages among the participants

A total of 206 M*.tb* DNA samples that were extracted from the TB culture positive specimens for spoligotyping (103 at baseline and 103 at follow-up). Spoligotyping data revealed that 82.52% (170/206) genotypes had distinct spoligotyping patterns: 20.59% (35/170) genotypes detected from the Hhohho region, 54.1% (92/170) from the Manzini region, the Lubombo and Shiselweni regions with 12.94% (22/170) and 12.35% (21/170) genotypes, respectively. Few specimens (*n* = 36/206) were labelled orphans as their spoligotype patterns were not found in the SITVIT2 database.

**Fig. 3:**
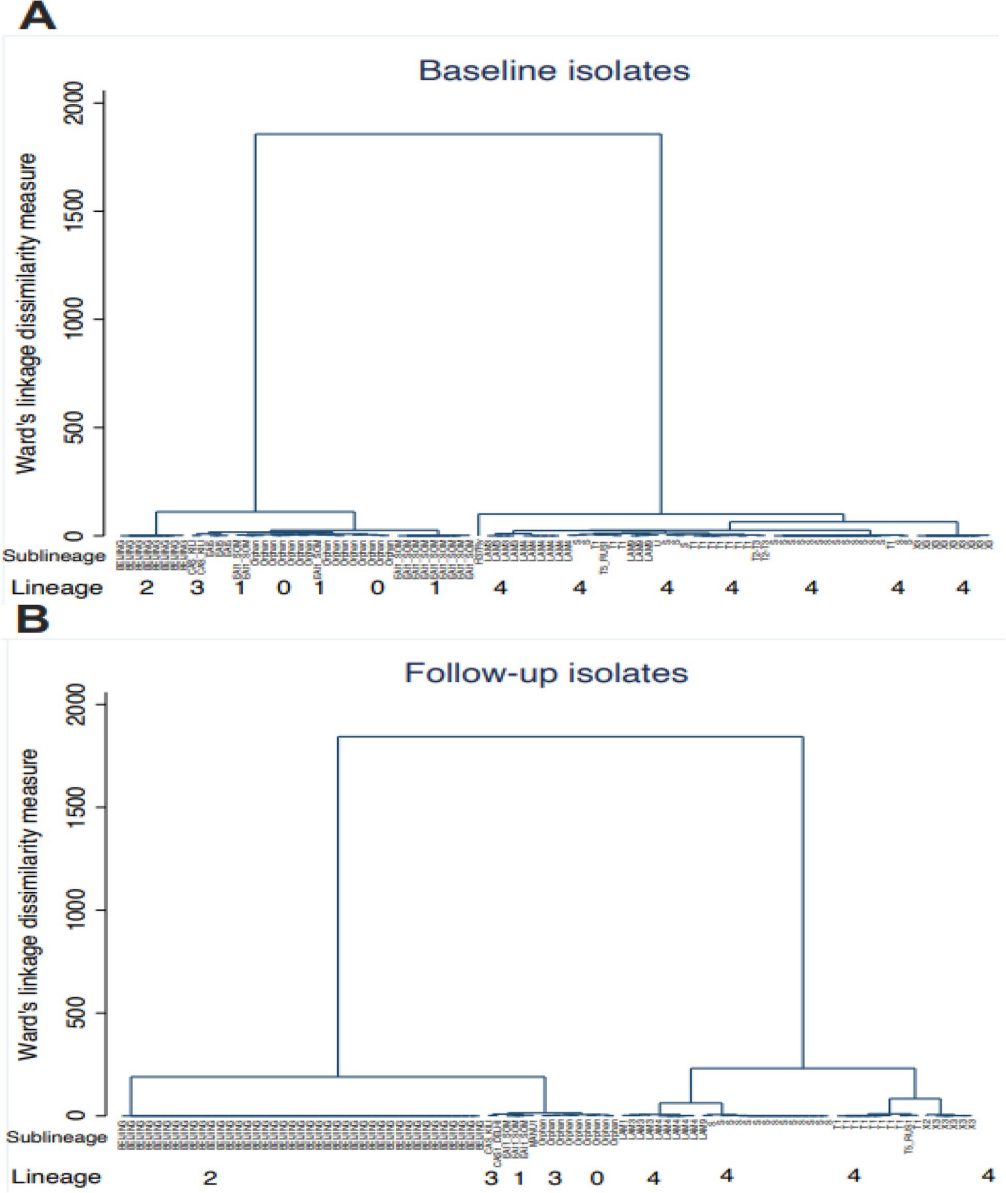
Spoligotype genotypes cluster analysis at baseline (diagram **A**) and follow up (diagram **B**). The figure shows the relationship between lineages and sublineages at baseline (diagram **A**) and follow-up (diagram **B**) to note clusters and measure of dissimilarity. Diagram (**A**) has 2 clusters, where the first cluster has lineage 1,3,1 and orphans and the second cluster is that of only lineage 4. The orphans are closely linked and similar to the lineage 1 EAI1_SOM and EAI5 clades. The second cluster of diagram (**A**) has only lineage 4 and simplicifolious in H37Rv sub lineage. The two clusters of diagram (**A**) have very small dissimilarities as the vertical heights are almost equal. The same observation on dissimilarity was made in diagram (**B**) which also two clusters. In the first cluster, lineage 2 has one clade compared to diagram (**A**) with two. The orphans are also linked to lineage 1 and 3 clades. The second cluster predominate sub lineage T1 has four clades which are close to X3 sub lineage. Comparing diagram (**A**) and (**B**), the measure of dissimilarity is higher in diagram (**B**)

The analyzed spoligotyping patterns revealed a total of four lineages: Indo-Oceanic (lineage 1), East Asian (lineage 2), Central Asian (lineage 3), and Euro-American (lineage 4) and twenty genotypes (EAI6-BGD1, EAI1_SOM, MANU1, MANU2, BEIJING, CAS_DELHI, CAS_KILI, T1, T2, S, X2, X3, LAM1, LAM3, LAM4, LAM5, LAM9, LAM11-ZWE, LAM-RUS,and T-H37Rv) among the study participants (Table 3) (Fig. 3).

**Table 3:** Detection of TB genotypes at baseline and follow-up among the study participants

Lineage	Lineage distribution n (%)	Genotype	Region								TOTAL
			**Hhohho n(%)**		**Manzini n(%)**		**Lubombo n(%)**		**Shiselweni n/13(%)**		**n(%)**
			Baseline	Follow-up	Baseline	Follow-up	Baseline	Follow-up	Baseline	Follow-up	
Indo-Oceanic (Lineage 1)	20 (9.71)	EAI6-BGD1	1 (4.76)	0 (0.00)	0 (0.00)	0 (0.00)	1 (7.69)	0 (0.00)	0 (0.00)	0 (0.00)	2 (0.97)
		EAI1-SOM	2 (9.52)	0 (0.00)	8 (14.29)	3 (5.36))	1 (7.69)	0 (0.00)	1 (7.69)	0 (0.00)	15 (7.28)
		Manu1	1 (4.76)	1 (4.76)	0 (0.00)	0 (0.00)	0 (0.00)	0 (0.00)	0 (0.00)	0 (0.00)	2 (0.97)
		Manu2	0 (0.00)	1 (4.76)	0 (0.00)	0 (0.00)	0 (0.00)	0 (0.00)	0 (0.00)	0 (0.00)	1 (0.49)
East Asian (Lineage 2)	50 (24.27)	BEIJING	2 (9.52)	8 (38.10)	4 (7.14)	25 (44.64)	2 (15.39)	6 (46.15)	1 (7.69)	2 (15.39)	50 (24.27)
Central Asian (Lineage 3)	4 (1.94)	CAS1-Delhi	0 (0.00)	0 (0.00)	0 (0.00)	0 (0.00)	0 (0.00)	0 (0.00)	0 (0.00)	1 (7.69)	1 (0.49)
		CAS1-Kili	1 (4.76)	0 (0.00)	1 (1.79)	0 (0.00)	0 (0.00)	1 (7.69)	0 (0.00)	0 (0.00)	3 (1.46)
Euro-American (Lineage 4)	95 (46.12)	X2	0 (0.00)	0 (0.00)	0 (0.00)	0 (0.00)	0 (0.00)	0 (0.00)	1 (7.69)	0 (0.00)	1 (0.49)
		X3	3 (14.29)	1 (4.76)	4 (7.14)	3 (5.36)	1 (7.69)	1 (7.69)	1 (7.69)	1 (7.69)	14 (6.80)
		LAM1	0 (0.00)	1 (4.76)	0 (0.00)	0 (0.00)	0 (0.00)	2 (15.39)	0 (0.00)	0 (0.00)	1 (0.49)
		LAM3	1 (4.76)	0 (0.00)	3 (5.36)	0 (0.00)	2 (15.39)	3 (23.08)	0 (0.00)	1 (7.69)	7 (3.40)
		LAM4	0 (0.00)	1 (4.76)	3 (5.36)	4 (7.14)	0 (0.00)	4 (30.77)	2 (15.39)	0 (0.00)	10 (4.85)
		LAM5	0 (0.00)	0 (0.00)	1 (1.79)	0 (0.00)	0 (0.00)	1 (7.69)	0 (0.00)	0 (0.00)	2 (0.97)
		LAM9	1 (4.76)	0 (0.00)	1 (1.79)	0 (0.00)	0 (0.00)	1 (7.69)	0 (0.00)	0 (0.00)	3 (1.46)
		LAM-RUS	0 (0.00)	0 (0.00)	0 (0.00)	1 (1.79)	1 (7.69))	0 (0.00)	0 (0.00)	0 (0.00)	2 (0.97)
		LAM11-ZWE	1 (4.76)	0 (0.00)	0 (0.00)	0 (0.00)	0 (0.00)	0 (0.00)	0 (0.00)	1 (7.69)	2 (0.97)
		S	5 (23.81)	3 (14.29)	9 (16.07)	3 (5.36)	3 (23.08)	3 (23.08)	3 (23.08)	5 (38.46)	34 (16.50)
		T1	0 (0.00)	3 (0.14)	7 (12.50)	5 (8.93)	1 (7.69)	0 (0.00)	0 (0.00)	1 (7.69)	17 (8.25)
		T2	0 (0.00)	0 (0.00)	1 (1.79)	0 (0.00)	0 (0.00)	0 (0.00)	0 (0.00)	0 (0.00)	1 (0.49)
		T-H37Rv	0 (0.00)	0 (0.00)	0 (0.00)	1 (1.79)	0 (0.00)	0 (0.00)	0 (0.00)	0 (0.00)	1 (0.49)
Orphan	37 (17.96)	Orphan	3 (0.14)	2 (0.1)	14 (25.00)	11 (19.64)	1 (7.69)	1 (7.69)	4 (30.77)	1 (7.69)	37 (17.96)
**TOTAL**			21 (10.19%)	21 (10.19%)	56 (27.18%)	56 (27.18%)	13 (6.31%)	13 (6.31%)	13 (6.31%)	13 (6.31%)	206 (100)

The distribution of the lineages varied among the different regions. The Euro-American (Lineage 4) was the most common lineage with 46.12% (95/206) followed by the East Asian (Lineage 2) with 24.27% (50/206) and 9.71% (20/206) for Indo-Oceanic (Lineage 1) and 1.94% (4/206) for the Central Asian (Lineage 3). Furthermore, a high proportion of the Beijing genotype at 24.27% (50/206) and S genotype at 16.50% (34/206). The S genotype was predominant in the Shiselweni region with 30% at baseline and at follow-up (27.3%) in Lubombo region. Furthermore, the Beijing genotype was predominant at baseline in the Hhohho region with 23.5% and Shiselweni had more of the Beijing genotypes at follow up with 54.5%.

Notably, in most regions there was a diversity of genotypes between baseline and follow-up specimens (see Supplementary material and Table 3). Some of genotypes at baseline were not detected at follow-up timepoint. In the Hhohho region lineage 1 genotypes (EAI1_SOM and EAI6-BGD1) and CAS-Kili, LAM11-ZWE, LAM3 and LAM9 genotypes were only present at baseline and missing at follow-up. Also, the Manzini region had the CAS1-Kili, LAM3 LAM4, LAM9 and T2 genotypes were only noted at baseline. Likewise, in the Shiselweni region the EAI1_SOM, LAM4 and X2 were only present at baseline. Other regions had some genotypes that emerged at follow-up specimens. This was found in Hhohho region (LAM1, LAM4 and Manu2 genotypes),Manzini region (LAM-RUS genotypes) and Shiselweni region (LAM11-ZWE genotype) with some genotypes appearing in follow-up specimens. Interestingly, the frequency of the Beijing genotype, was observed to be highly present at follow-up specimens in most regions.

The Hhohho and Manzini regions had high diversity of TB genotypes, followed by the Lubombo region (*n* = 11/20) and Shiselweni region (*n* = 10/20). Some lineages had decreased frequency at follow-up compared to baseline. These were X3 and S genotypes in the Hhohho region; the LAM4 genotype in the Shiselweni region; EAI1_SOM, EAI6-BGD1 and S genotypes in the Manzini region. The same was noted in Lubombo region with the EAI1_SOM, EAI6-BGD1, X3, LAM3 and LAM-RUS genotypes. Moreover, some genotypes had the same frequencies between baseline and follow-up. The Beijing genotype was the most prevalent in follow-up specimens.

**Fig. 4:**
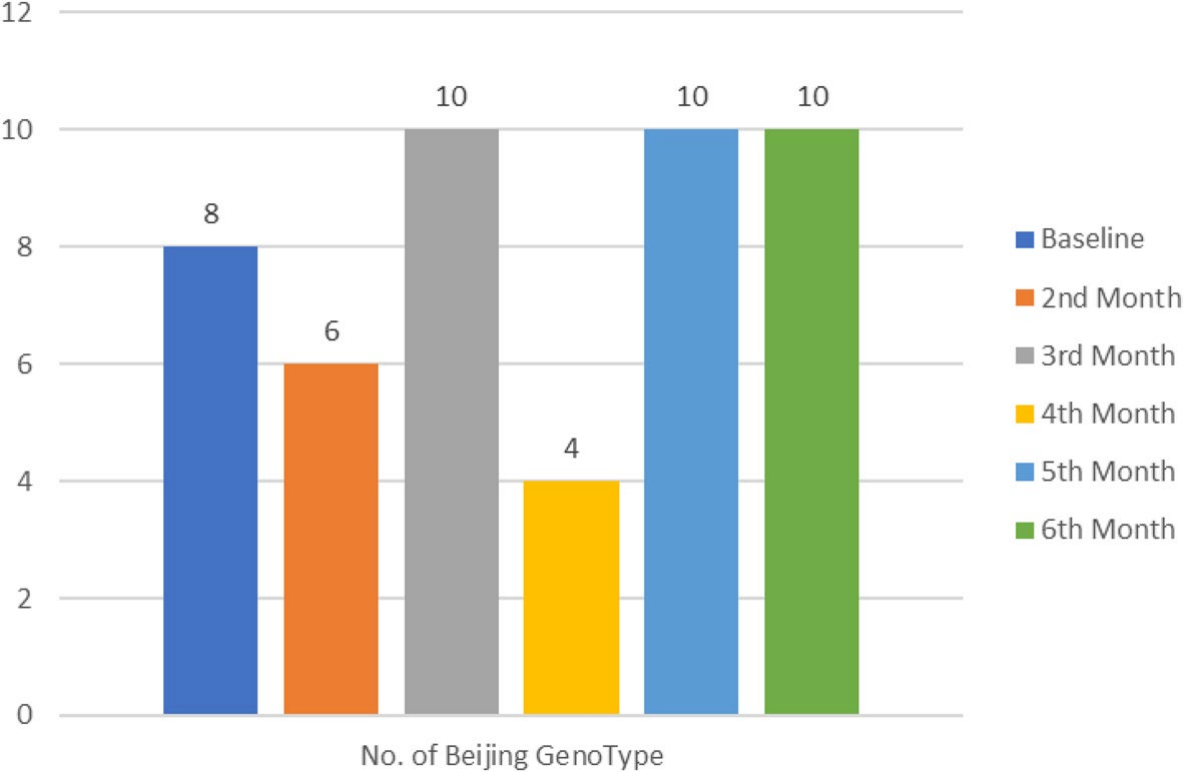
Detection of Beijing genotypes in serial isolates. At follow up, most Beijing strains were isolated on the 3^rd^, 5^th^ and 6^th^ month. The least were detected in the 4^th^ month

Further investigation reveled that some susceptible *M.tb* specimens later developed into MDR-TB and in the process acquired a completely new strain at follow-up timepoint. Comparison of both baseline (start of treatment) and follow-up genotypic data at six months or the last available positive specimen from patients on treatment was done. Our results showed that a significant proportion of follow-up specimens developed into MDR-TB (*p* = 0.0014) (Table 4) and Beijing was mostly detected in follow-up specimens (*p* < 0.000) (Table 5). Most of the Beijing genotypes were detected on third, fifth and sixth month (Fig. 4).

**Table 4:** Association MDR-TB development with follow-up specimens

Characteristic		Total n (%)	MDR-TB n (%)	Non-MDR TB n (%)	*P value
Total		206 (100%)	60 (29.2%)	146 (70.8%)	
Specimen type	Baseline	103 (50%)	22 (21.4%)	81 (78.6%)	0.0014*
	Follow-up	103 (50%)	38 (36.9%)	65 (63.1%)	

^*^Pearson Chi (square)

**Table 5:** Detection of Beijing genotype in follow-up specimens

Characteristic		Total	Beijing	Non-Beijing n	*P* value
Total		^a^195 (100%)	97 (49.7%)	98 (50.3%)	
Spoligotype	Beijing	48 (24.6%)	40 (83.3%)	8 (16.7%)	< 0.0000*
	Non-Beijing	147 (75.4%)	57 (38.8%)	90 (61.2%)	

^*^Pearson Chi (square)

^a^Twelve (12) specimens had orphan results at baseline (*n* = 5) and or follow-up (*n* = 7)

Mixed-strain TB infection refers to TB disease caused by more than one clonally distinct *M.tb* strain, either through a single transmission event involving more than one distinct strain or through multiple transmission events (super-infection) during a single disease episode [12]. The study detected high number of isolates (64.5%; *n* = 133/206) that developed mixed-strain infections during treatment. The Beijing genotype had the highest number of mixed-strain infections, followed by S and T1 genotypes (Table 6). A total of 35 (23.3%) mixed-strain infections were resistant to all four first line drugs (Table 7).

**Table 6:** The distribution of TB genotypes among single and mixed strains

Sub-lineage	Single strain n (%)	Mixed strain n (%)	Total n (%)
EAI6-BGD1	2 (0.97)	0 (0.0)	2 (0.97)
EAI1-SOM	2 (0.97)	13 (6.31)	15 (7.28)
Manu1	2 (0.97)	0 (0.0)	2 (0.97)
Manu2	1 (0.49)	0 (0.0)	1 (0.49)
BEIJING	15 (7.28)	35 (16.99)	50 (24.27)
CAS1-Delhi	0 (0.0)	1 (0.49)	1 (0.49)
CAS1-Kili	1 (0.49)	2 (0.97)	3 (1.46)
X2	0 (0.0)	1 (0.49)	1 (0.49)
X3	2 (0.97)	12 (5.83)	14 (6.80)
LAM1	0 (0.0)	1 (0.49)	1 (0.49)
LAM3	0 (0.0)	7 (3.40)	7 (3.40)
LAM4	3 (1.46)	7 (3.40)	10 (4.85)
LAM5	2 (0.97)	0 (0.0)	2 (0.97)
LAM9	2 (0.97)	1 (0.49)	3 (1.46)
LAM-RUS	0	2 (0.97)	2 (0.97)
LAM11-ZWE	1 (0.49)	1 (0.49)	2 (0.97)
S	28 (13.59)	6 (2.91)	34 (16.50)
T1	11 (5.34)	6 (2.91)	17 (8.25)
T2	1 (0.49)	0 (0.0)	1 (0.49)
T-H37Rv	1 (0.49)	0 (0.0)	1 (0.49)
Orphan	0 (0.0)	37 (17.96)	37 (17.96)
TOTAL	73 (35.44%)	133 (64.56)	206 (100)

**Table 7:** The distribution of drug resistant patterns among single and mixed strains

RIF/INH/STR/EMB	Single strain n (%)	Mixed strain n (%)	Total n (%)
RRRR	6 (8.22)	41 (30.83)	47 (22.82)
RRRS	0 (0.00)	3 (2.26)	3 (1.46)
RRSR	0 (0.00)	5 (3.76)	5 (2.43)
RRSS	0 (0.00)	3 (2.26)	3 (1.46)
RSRR	3 (4.11)	1 (0.75)	4 (1.94)
RSRS	8 (10.96)	2 (1.50)	10 (4.85)
RSSR	3 (4.11)	0 (0.00)	3 (1.46)
RSSS	4 (5.48)	2 (1.50)	6 (2.91)
SRRS	0 (0.00)	3 (2.26)	3 (1.46)
SRSS	1 (1.37)	3 (2.26)	4 (1.94)
SSRS	1 (1.37)	0 (0.00)	1 (0.49)
SSSS	47 (64.38)	70 (52.62)	117 (56.80)
Total	73 (100)	133	206 (100)

## Discussion

The study investigated the TB genotypes circulating in Eswatini using spoligotyping on specimens collected at baseline (start of treatment) and follow-up with resistant data at six months or the last available positive specimen from patients on treatment. TB cases in Eswatini are high and increased transmission in communities, together with ineffective drugs may drive infection rates [13]. The TB drug resistance survey conducted in Eswatini in 2012 reported an MDR-TB prevalence of 33.8% [14]. Similarly, to this study, males continue to drive the TB infection in the country [15]. This suggests that males could be the main transmitters of TB and considered to be a high-risk group. Of concern is the fact that males have greater social contacts; spend extra time in settings that may be conducive to transmission (i.e. consisting of bars) and have interaction in professions associated with acquisition of TB, such as mining [16].

Several studies including those in the WHO Africa region concur with the current study findings, showing high TB incidence among adolescents and young adults [15]. Also, a study done in South Africa, showed the highest burden of TB in young adults [17]. In Eswatini, young adults contributes to approximately 27% of the total population who are active employment seekers [18]. Socio-cultural norms on gender roles associate men’s role in the household economy with limited employment for women [18]. When males are infected with TB, there may be loss of income in their households due to frequent travel to health centres for treatment, increased need for nutrition, hospitalisation and worse if there is a death [18]. Therefore, it is important that TB is detected and treated timeously among residents of Eswatini.

MDR-TB was highly detected in Lubombo region, together with RIF mono-resistance with 50.0% followed by the INH mono-resistance at 42.3%. It is well documented that resistance to these two drugs is used as markers for MDR-TB [4]. These findings of significant proportions of MDR-TB in this region may probably be due to poor TB control issues in rural settings. Eswatini rural settings have been associated with development of MDR-TB. A study conducted in rural South African northern districts of KwaZulu-Natal found the highest rate of MDR-TB cases [19]. This highlights that management of TB in rural settings is important and requires special attention for successful treatment program.

Further analysis of the findings on the resistance to anti-TB drugs highlighted that 41.26% participants had mono-drug resistance (i.e. 28.61% of RIF mono-resistance), some participants had poly-resistance at 8.25% which may indicate that a proportion of TB infected patients in Eswatini may be resistant to at least one of the first line anti-TB drugs. Similarly to a study conducted in Eswatini among previously treated patients Sanchez-Padilla, Dlamini [14]. Many cases of mono-resistant TB may contribute to the amplification of resistance and ultimately result in MDR-TB [20]. The standardized first line TB drug regimen in Eswatini is adopted from WHO and consists of two phases (intensive and continuation phase) [4].

Our study provided the most recent insights into the genetic diversity of *M.tb* genotypes circulating in Eswatini. Of the seven known *M.tb* lineages [21], the observed *M.tb* genotypes in the current study belonged to lineages one to four. The Euro-American lineage (lineage 4) was the most common lineage in the study, followed by the East Asian lineage (lineage 2), Indo-Oceanic lineage (lineage 1) and Central Asian lineage (lineage 3). The Beijing genotype was the predominant, followed by the S and T1 genotypes (lineage 4) in 20.0% and 11.76% of specimens, respectively. The lineage heterogeneity in Eswatini may potentially render a challenge in putting in place an effective TB control programme, which would control the spread of the TB infection in communities [22].

All TB lineages have different virulence factors which may have effects on the anti-TB treatment outcomes. It is generally accepted that the overall strain success to survive treatment effects relies upon the combination of strain virulence traits and host genetic elements [4]. Hence lineage diversity might be a contributing factor towards the development of MDR-TB and treatment failure. Most importantly Beijing genotype which is widely common among MDR-TB outbreaks may be associated with the high MDR-TB rate in Eswatini. Further analysis revealed that the proportion of participants with MDR-TB was high in follow-up specimens. The regions of Manzini and Shiselweni experienced significant increases in participants infected with the Beijing genotype. The Beijing genotype is mainly associated with high prevalence of MDR-TB [23]. The Beijing genotype was the second most prevalent after the S genotype in the Lubombo region, and this region had the highest rate of MDR-TB at 38.46%. The second region with high MDR-TB was the Hhohho with 30.95% with high levels of the Beijing genotype. These findings support the suggestion that the Beijing genotype is related to excessive occurrence of drug resistant TB [24]. Most of the isolated Beijing genotypes were significantly detected in follow-up specimens, which may indicate super-infection when the severity of a current disease episode is such that it compromises the host innate immune response to a point that leads to increased susceptibility to infection with a secondary strain [25]. Our population has a high prevalence of HIV, although it was not tested, this may indicate low protective immune response against TB which may be prone to secondary infections.

The X2 and CAS-Delhi genotypes were detected in Shiselweni region. The X2 genotype is commonly detected in the Americas population and other countries of Southern Africa, Asia, and Europe [26]. The occurrence of X2 genotype might have been acquired in Eswatini through increased migration of people from different countries where it is dominant [26]. CAS-Delhi genotypes are commonly isolated in the Middle East and Central Asia. It has also been found in regions with frequent migration to and from the Indian subcontinent [27]. Furthermore, the Manu1, Manu2, T2 and LAM1 genotypes were found in few participants from the Hhohho and Manzini regions that are considered urban regions. The Manu genotypes were isolated in high proportion from Egyptian patients in Africa [28]. However, the MANU2 family has a low prevalence worldwide and few specimens in the current study had this genotype.

Furthermore, CAS-Delhi and CAS-Kili (lineage 3) genotypes are associated with increased relapse and reinfection rates among TB infected patients [29]. According to Eswatini (formerly Swaziland National Tuberculosis Control Program [2], the Lubombo region had 5% TB infection relapse rate while both Hhohho and Manzini region had 2% with Shiselweni at 1% relapse rate. Therefore, based on Guerra-Assunção, Houben [30] findings, the current study may point to the lineage 3 genotypes as a possible cause of relapse cases in the Hhohho, Shiselweni and the Lubombo regions of Eswatini.

In this present study, most patients had multiple or mixed-strain genotypes either at baseline or follow-up. This identification of mixed-strain infection, which is an infection with several different genotypes of *M.tb* in a single patient during the course of treatment was an interesting finding [31]. Sputum specimens and typing methods may under-estimate the true bacterial diversity within the lung as they are likely limited to reveal the coexistence of multiple *M.tb* strains in the same patient [32]. In this study, the Beijing genotype had a substantial rate of mixed-strain genotypes found in 35/206 (16.99%) specimens and the S genotype at 28/206 (13.59%) in single strain infection. Previous studies in the African continent, reported that Beijing and S genotypes are most likely to be associated with mixed infections [33, 34]. Changing drug-susceptibility patterns is common among mixed-strain infections during therapy. Mixed-strain infection with both drug sensitive and resistant profiles may lead to discordant drug-susceptibility testing profiles, which could complicate the treatment regimen and lead to poor treatment outcomes [5]. The mixed-strain infections could have arised, if the inital infectious episode at baseline, caused by a distinct strain, could results in relapse of the original infection, yielding disease with two unique *M.tb* strains that may have the same or different drug susceptibility profiles [33]. The detection of the mixed-strain infection in the study is an important finding and has an impact on success of patient treatment. Moreover, treatment could have reduced the initial drug susceptible population while allowing the drug resistant population to grow and cause drug resistance.Further analysis revealed that some participants who had drug resistant genotypes at baseline or at follow-up, had genotype and/or DST profile change between baseline and follow-up episodes. A total of 33 (53.23%) participants had mixed-strain infections with the same drug sensitivity pattern between baseline and follow-up, followed by 16 (25.81%) participants with mixed-strain infection with different drug sensitivity patterns. This was concerning as these findings were observed at six months after the administration of the conventional first line drug regimen, which was expected to subside the infection rate. Similarly, twenty-four of 37 (64.9%) patients with serial culture positives showed different genotypes at different time points and changes in the genotype while on treatment was a very important observation. It is reported that genetic changes promote attainment of autonomous pathogenic traits within TB lineages, and thus confers resistance to some anti-TB drugs [12]. Thus, it is important to report the nature of mixed-strain infection in populations of high burden countries, especially in studies with large cohorts.

Due to the fact that we could only follow the participants for six months and had low sample size, the results could not allow the generalisation of the findings to the general population of Eswatini. However, the findings provide a snapshot of TB strain lineages circulating in Eswatini and their DST patterns. The detection of high mixed infections requires further investigation with highly sensitive methods (i.e. MIRU VNTR). Despite the study limitations, the study provides the population structure of genotypes circulating in four regions of Eswatini.

## Conclusion

Ewatini has a high TB lineage diversity which may potentially render a challenge in implementing an effective TB control programme. Therefore various genotypes of *M.tb* strains circulating in Eswatini needs to be investigated and monitored to improve our understanding the TB transmission dynamics of these clones in the geographic regions during treatment. Mixed strain infections could lead to poor treatment outcomes and potentially lead to development of drug resistant TB genotypes. Therefore, to improve TB treatment outcomes in Eswatini, it is recommended that the Ministry of Health reviews their TB drug susceptibility testing guidelines, to allow for typing of strains in the patients diagnosed with TB at least twice before the completion of treatment.

### Supplementary Information

**Additional file 1: Supplementary Material.** Baseline findings. **Supplementary Material.** Follow-up findings.

## Data Availability

The datasets used and/or analysed during the current study are available from the corresponding author on reasonable request.

## Abbreviations

DST: Drug susceptibility testing
EMB: Ethambutol
INH: Isoniazid
LPA: Line probe assay
MDR-TB: Multidrug resistant tuberculosis
MGIT: Mycobacterium growth indicator tube
RIF: Rifampicin
WHO: World health organization
ZN: Ziehl–Neelsen
PZA: Pyrazinamide
DR: Drug resistant
