# Supplementary material for: Molecular investigations of Mycobacterium tuberculosis genotypes among baseline and follow-up strains circulating in four regions of Eswatini
Source: BMC Infect Dis. 2023 Aug 29;23:566. doi: 10.1186/s12879-023-08546-9 (PMC10466871; doi:10.1186/s12879-023-08546-9)
Supplement: Supplementary file 1 — Additional file 1: Supplementary Material. Baseline findings. Supplementary Material. Follow-up findings. [file 12879_2023_8546_MOESM1_ESM.docx]

**Supplementary Material: Baseline findings**

| Lineages | Sub-lineage | DST PATTERN | | | | n | MDR | Non-MDR | Total (%) |
| --- | --- | --- | --- | --- | --- | --- | --- | --- | --- |
|  |  | RIF | INH | STR | EMB |  |  |  |  |
| Lineage 1 | EAI1_SOM | S | S | S | S | 7 | 0 | 7 | 15 (14.56) |
|  |  | R | R | R | R | 2 | 2 | 0 |  |
|  |  | R | S | R | R | 1 | 0 | 1 |  |
|  |  | S | R | R | S | 1 | 0 | 1 |  |
|  |  | S | R | S | S | 1 | 0 | 1 |  |
|  | EAI5 | S | S | S | S | 2 | 0 | 2 |  |
|  |  | R | S | S | S | 1 | 0 | 1 |  |
| Subtotal 1 | | | | | | 15 | 2 | 13 |  |
| Lineage 2 | Beijing | S | S | S | S | 6 | 0 | 6 | 7 (6.79) |
|  |  | R | R | R | R | 1 | 1 | 0 |  |
|  |  | S | S | R | S | 1 | 0 | 1 |  |
| Subtotal 2 | | | | | | 8 | 1 | 7 |  |
| Lineage 3 | CAS_KILI | S | S | S | S | 1 | 0 | 1 | 2 (1.94) |
|  |  | R | R | R | R | 1 | 1 | 0 |  |
| Subtotal 3 | | | | | | 2 | 1 | 1 |  |
| Lineage 4 | T1 | S | S | S | S | 7 | 0 | 7 | 58 (56.31) |
|  |  | R | R | R | R | 2 | 2 | 0 |  |
|  |  | R | S | S | R | 1 | 0 | 1 |  |
|  |  | S | R | S | S | 1 | 0 | 1 |  |
|  | T5_RUS1 | R | R | R | S | 1 | 0 | 1 |  |
|  | T1_T3 | R | R | R | R | 1 | 0 | 1 |  |
|  |  | S | R | S | S | 1 | 0 | 1 |  |
|  | S | S | S | S | S | 13 | 0 | 13 |  |
|  |  | R | R | R | R | 3 | 3 | 0 |  |
|  |  | R | R | S | S | 1 | 1 | 0 |  |
|  |  | R | S | R | S | 2 | 0 | 2 |  |
|  |  | R | S | S | R | 1 | 0 | 1 |  |
|  | X2 |  |  |  |  |  | 0 | 0 |  |
|  | X3 | S | S | S | S | 6 | 0 | 6 |  |
|  |  | R | R | R | R | 1 | 1 | 0 |  |
|  |  | R | S | R | S | 1 | 0 | 1 |  |
|  |  | R | S | S | S | 1 | 0 | 1 |  |
|  | U | S | S | S | S | 1 | 0 | 1 |  |
|  | LAM1 |  |  |  |  |  | 0 | 0 |  |
|  | LAM3 | S | S | S | S | 4 | 0 | 4 |  |
|  | LAM4 | S | S | S | S | 3 | 0 | 3 |  |
|  |  | R | R | R | R | 1 | 1 | 0 |  |
|  |  | R | S | R | S | 1 | 0 | 1 |  |
|  |  | R | S | S | R | 1 | 0 | 1 |  |
|  | LAM9 | S | S | S | S | 3 | 0 | 3 |  |
|  | H37RV | R | S | R | S | 1 | 0 | 1 |  |
| Subtotal 4 | | | | | | 58 | 8 | 50 |  |
| Orphan | | S | S | S | S | 9 | 0 | 9 | 15 (14.56) |
|  |  | R | R | R | R | 4 | 4 | 0 |  |
|  |  | R | R | S | R | 1 | 1 | 0 |  |
|  |  | R | S | S | S | 1 | 0 | 1 |  |
| Subtotal | | | | | | 15 | 5 | 10 |  |
| Negative | | S | S | S | S | 4 | 0 | 4 | 5 (4.85) |
|  |  | R | R | R | R | 1 | 1 | 0 |  |
| Subtotal | | | | | | 5 | 1 | 4 |  |
| Grand total | | | | | | 103 | 18 | 85 | 103 (100) |

**Supplementary Material: Follow-up findings**

| Lineages | Sub-lineage | DST PATTERN | | | | n | MDR | Non-MDR | Total (%) |
| --- | --- | --- | --- | --- | --- | --- | --- | --- | --- |
|  |  | RIF | INH | STR | EMB |  |  |  |  |
| Lineage 1 | EAI1_SOM | S | S | S | S | 1 | 0 | 1 | 4 (3.88) |
|  |  | R | R | R | R | 2 | 2 | 0 |  |
|  | MANU1 | S | S | S | S | 1 | 0 | 1 |  |
| Subtotal 1 | | | | | | 4 | 2 | 2 |  |
| Lineage 2 | Beijing | S | S | S | S | 22 | 0 | 22 | 41 (39.81) |
|  |  | R | R | R | R | 14 | 14 | 0 |  |
|  |  | R | R | S | R | 1 | 1 | 0 |  |
|  |  | R | S | S | S | 1 | 0 | 1 |  |
|  |  | R | S | R | R | 1 | 0 | 1 |  |
|  |  | R | S | R | S | 1 | 0 | 1 |  |
|  |  | S | R | S | S | 1 | 0 | 1 |  |
| Subtotal 2 | | | | | | 41 | 15 | 26 |  |
| Lineage 3 | CAS_DELHI | S | S | S | S | 1 | 0 | 1 | 2 (1.94) |
|  | CAS_KILI | R | R | S | R | 1 | 1 | 0 |  |
| Subtotal 3 | | | | | | 2 | 1 | 1 |  |
| Lineage 4 | T1 | S | S | S | S | 3 | 0 | 3 | 40 (38.83) |
|  |  | R | R | R | R | 1 | 1 | 0 |  |
|  |  | R | R | R | S | 1 | 1 | 0 |  |
|  |  | R | R | S | R | 1 | 1 | 0 |  |
|  |  | R | S | S | S | 1 | 0 | 1 |  |
|  |  | R | S | R | R | 1 | 0 | 1 |  |
|  |  | R | S | R | S | 1 | 0 | 1 |  |
|  | T5_RUS1 | S | R | R | S | 1 | 0 | 1 |  |
|  | S | S | S | S | S | 7 | 0 | 7 |  |
|  |  | R | R | R | R | 4 | 4 | 0 |  |
|  |  | R | R | S | S | 1 | 1 | 0 |  |
|  |  | R | S | R | S | 2 | 0 | 2 |  |
|  | X2 | R | R | R | S | 1 | 1 | 0 |  |
|  | X3 | S | S | S | S | 2 | 0 | 2 |  |
|  |  | R | R | R | R | 1 | 1 | 0 |  |
|  |  | R | S | R | R | 2 | 0 | 2 |  |
|  | LAM1 | R | R | R | R | 1 | 1 | 0 |  |
|  | LAM3 | S | S | S | S | 2 | 0 | 2 |  |
|  |  | R | S | S | R | 1 | 0 | 1 |  |
|  | LAM4 | S | S | S | S | 5 | 0 | 5 |  |
|  | LAM9 | R | R | R | R | 1 | 1 | 0 |  |
| Subtotal 4 | | | | | | 40 | 12 | 28 |  |
| Orphan | | S | S | S | S | 2 | 0 | 2 | 9 (8.74) |
|  |  | R | R | R | R | 6 | 6 | 0 |  |
|  |  | R | S | R | S | 1 | 0 | 1 |  |
| Subtotal | | | | | | 9 | 6 | 3 |  |
| Negative | | S | S | S | S | 5 | 0 | 5 | 7 (6.80) |
|  |  | R | R | S | S | 1 | 1 | 0 |  |
|  | | R | S | S | S | 1 | 0 | 1 |  |
| Subtotal | | | | | | 7 | 1 | 6 |  |
| Grand total | | | | | | 103 | 37 | 66 | 103 (100) |
